# Non-canonical Roles of Telomerase: Unraveling the Imbroglio

**DOI:** 10.3389/fcell.2019.00332

**Published:** 2019-12-10

**Authors:** Evelyne Ségal-Bendirdjian, Vincent Geli

**Affiliations:** ^1^INSERM UMR-S 1124, Team: Cellular Homeostasis, Cancer and Therapies, INSERM US36, CNRS UMS 2009, BioMedTech Facilities, Université de Paris, Paris, France; ^2^Marseille Cancer Research Center, U1068 INSERM, UMR 7258 CNRS, Aix Marseille University, Institut Paoli-Calmettes, Equipe labellisée Ligue, Marseille, France

**Keywords:** telomere, telomerase (TERT), signaling pathway, stem cell function, mitochondria

## Abstract

Telomerase plays a critical role in stem cell function and tissue regeneration that depends on its ability to elongate telomeres. For nearly two decades, it turned out that TERT regulates a broad spectrum of functions including signal transduction, gene expression regulation, and protection against oxidative damage that are independent of its telomere elongation activity. These conclusions that were mainly obtained in cell lines overexpressing telomerase were further strengthened by *in vivo* models of ectopic expression of telomerase or models of G1 *TERT* knockout mice without detectable telomere dysfunction. However, the later models were questioned due to the presence of aberrantly shortened telomere in the germline of the parents *TERT^+/–^* that were used to create the G1 *TERT*^–/–^ mice. The physiological relevance of the functions associated with overexpressed telomerase raised also some concerns due to artifactual situations and localizations and complications to quantify the level of TERT. Another concern with non-canonical functions of TERT was the difficulty to separate a direct TERT-related function from secondary effects. Despite these concerns, more and more evidence accumulates for non-canonical roles of telomerase that are non-obligatory extra-telomeric. Here, we review these non-canonical roles of the TERT subunit of telomerase. Also, we emphasize recent results that link TERT to mitochondria and protection to reactive oxygen species suggesting a protective role of TERT in neurons. Throughout this review, we dissect some controversies regarding the non-canonical functions of telomerase and provide some insights to explain these discrepancies. Finally, we discuss the importance of understanding these alternative functions of telomerase for the development of anticancer strategies.

## Introduction

Mammalian telomeres are specialized DNA and protein structures found at the very ends of linear chromosomes. They consist of hexameric DNA 5′-TTAGGG*_n_* repeats associated with a specific shelterin complex of six proteins and are essential for chromosomal maintenance and genomic stability (de Lange, 2018). Telomeres shorten when normal cells undergo each replication due to the “end replication problem,” resulting mostly from incomplete lagging-strand DNA synthesis and the induction of exonucleolytic processing at the leading strand (Lingner et al., 1995; Faure et al., 2010; Lam et al., 2010; Wu et al., 2012). Telomere shortening is compensated by telomerase, a specialized ribonucleoprotein (RNP) complex that contains at least two major components and several accessory proteins (Shay and Wright, 2019). The first major component is a protein with reverse transcriptase (RT) activity, the human telomerase RT (hTERT). This enzyme extends the telomeric DNA by adding short repetitive DNA sequences. The other component is a functional RNA, the human telomerase RNA (*hTR*), which functions as a template to direct the synthesis of telomeric repeats by hTERT. The complex is associated with accessory proteins such as dyskerin (DKC1), telomerase Cajal body protein 1 (TCAB1), non-histone chromosome protein 2 (NHP2), nucleolar protein 10 (NOP10), and GAR1 RNP (GAR1). Telomerase homeostasis depends highly on the coordinated regulation of telomerase RNP assembly, trafficking, and recruitment to telomeres (Schmidt and Cech, 2015).

Telomerase is expressed in adult stem cells, including skin, intestines, and hematopoietic stem/progenitor cells. Telomerase is transiently activated only in certain proliferating stem-like cells, such as T cell upon activation. However, upon differentiation, this transient activation goes off (Holt et al., 1996; Greenberg et al., 1998). The mechanisms of this transient activation and subsequent repression upon differentiation remained yet unknown. In cancer cells, the situation is different since telomerase that counteracts telomere shortening and senescence is reactivated (Bodnar et al., 1998). Telomerase activation occurs in 85–90% of all human cancers (Kim et al., 1994; Shay and Bacchetti, 1997) while 10–15% of cancers maintain their telomeres through a DNA recombination pathway (named ALT for alternative lengthening of telomeres) (Bryan et al., 1997). Reactivation of telomerase activity is mainly dependent on the increased expression of the *hTERT* gene (Yamaguchi et al., 2003) that appears to be the limiting component in cancer cells for the formation of the active enzyme. However, even though *hTR* is broadly expressed in normal cells (Feng et al., 1995), it is also often deregulated during tumorigenesis (Soder et al., 1997, 1998; Heine et al., 1998; Yamaguchi et al., 2003). Different estimations of the endogenous levels of *hTR* and hTERT protein and the assembled telomerase RNP were reported (Yi et al., 2001; Cohen et al., 2007; Xi and Cech, 2014). However, telomerase quantification was hampered by the difficulty to detect low level of the endogenous protein and also by technical limitations (see below). Studies using both RT-qPCR and northern report that *hTR* levels were often in excess over telomerase RNP complexes in cancer cells (Xi and Cech, 2014) (approximately 1150 *hTR* molecules in HeLa cells, whereas only approximately 500 molecules of hTERT) suggesting the existence of unassembled components. Therefore, a pool of hTERT-free *hTR* might assemble with other protein components and could demonstrate alternative functions independent of telomerase in cell survival and apoptosis as mentioned by some reports (Kedde et al., 2006; Gazzaniga and Blackburn, 2014). In the same vein, in HEK293 and HeLa approximately 240 telomerase complexes per cell have been estimated suggesting that free hTERT protein may not be assembled to *hTR* raising the question of independent functions of *hTR* and hTERT. As hTERT has a favored affinity for RNA, it is likely to interact with other long non-coding RNA than *hTR* (Nelson and Shippen, 2015; El Hajj et al., 2018). This possibility of hTERT interaction with non-conventional partners (not only proteins but also RNAs) provides interesting new insights into extratelomeric functions of telomerase.

Indeed, in the last 15 years it appeared that telomerase functions could not be restricted to telomeres and the list of telomere-unrelated functions progressively increased. Increasing studies reported a wider spectrum of telomerase functions including signal transduction pathways, gene expression regulation, and mitochondrial function with consequences on control cell survival, proliferation, differentiation, migration, and regeneration (Passos et al., 2007; Martinez and Blasco, 2011; Chiodi and Mondello, 2012; Li and Tergaonkar, 2014; Zhou et al., 2014; Miwa and Saretzki, 2017). These non-canonical functions of telomerase appear not only in mammals but have been also discovered in zebrafish (Imamura et al., 2008; Alcaraz-Perez et al., 2014). However, even though evidence indicates that telomerase elicits other functions, it is not excluded that some of the consequences associated with telomerase expression even if they are not related to telomerase elongation function, may be explained by functions at telomeres such as protective functions or telomere chromatin regulation. Some of them necessitate the RT domain, while some are *TR* independent. In this review, we briefly described the consequences of the critical shortening of telomeres and then summarize and discuss the proposed non-canonical roles of telomerase in particular in the context of its reactivation in cancer. Since many excellent reviews have been published on the extra-telomeric functions of TERT (Ding et al., 2013; Li and Tergaonkar, 2014; Saretzki, 2014; Maida and Masutomi, 2015; Teichroeb et al., 2016; Romaniuk et al., 2019; Yuan and Xu, 2019), the main purpose of this review will be to discuss some discrepancies found in the literature regarding the non-canonical functions of telomerase and discuss how can we explain these differences.

## The Many Faces of the Response to Telomere Dysfunction

The main pathway in response to telomere erosion is the p53-dependent tumor suppressive mechanism that downregulates the expression of several factors involved not only in cell cycle control but also in DNA repair and telomere maintenance (Vogelstein et al., 2000). This transcriptional repression is coordinated by the p21–DREAM complex that acts downstream p53 and binds E2F and cell cycle genes homology region (CHR) elements (Engeland, 2018). Activation of p53 in response to telomere dysfunction is also associated with defective mitochondrial biogenesis through repression of peroxisome proliferator-activated receptor gamma co-activator 1 alpha/beta (PGC-1α/β) (Sahin et al., 2011) and downregulation of all sirtuins including mitochondrial sirtuins Sirt3, 4, and 5 further extending the links between short telomeres and metabolic control (Amano et al., 2019). Besides, changes in telomere structure due to telomere shortening may release telomeric factors such as Rap1 and TRF2 that can associate with non-telomeric sites (Martinez et al., 2010; Yang et al., 2011; Ye et al., 2014). Such binding of telomeric factors outside telomeric regions can influence the transcription of genes involved in processes as different as metabolism, immunity, and differentiation (Martínez et al., 2013; Yeung et al., 2013; Cherfils-Vicini et al., 2019; Zizza et al., 2019). Along the same line, the shelterin protein TIN2 can be post-translationally processed in mitochondria and promotes oxidative phosphorylation unveiling unsuspected links between a shelterin protein and ROS production (Chen et al., 2012). In addition, short telomeres can trigger epigenetic changes at telomeric and subtelomeric chromatin and in TElomeric Repeat-containing RNA (TERRA) transcription that may perturb cell homeostasis (Porro et al., 2014a, b; Tardat and Déjardin, 2018). These results illustrate the complex response to telomere erosion and the intimate relationship between telomere attrition and exhaustion of proliferative potential, metabolic control, differentiation, and reduced lifespan. This was exemplified *in vivo* by the fact that the degenerative phenotypes, including neurodegeneration of mice with severe telomere dysfunction associated with repressed mouse *TERT* (*mTERT*) expression, were rescued by reactivating the expression of telomerase (Jaskelioff et al., 2011).

## Non-Telomeric Telomerase Activities in Signaling Pathways

Telomerase was first recognized for its telomere-lengthening activity. However, it is now well established that TERT participates in a variety of biological pathways independently of its telomere-lengthening function. These pathways are involved both in physiological processes including stem cell function, tissue homeostasis, and aging, and oncogenesis by promoting many features involved in tumorigenesis, tumor progression, and resistance to treatments. TERT is now well recognized to act as a transcription (co-)factor to regulate gene expression. Some examples are discussed below, the two most-studied signaling pathways regulated by hTERT being the Wnt/β-catenin and the nuclear factor kappa-light-chain-enhancer of activated B cells (NF-κB) pathways.

### TERT and the Wnt/β-Catenin Pathway: Autopsy of a Controversy

*In vivo* studies carried out in mice lay out the premises for extratelomeric roles of TERT in tumorigenesis. *mTERT* expression at high levels in transgenic mice (associated with an increased telomerase activity) in several tissues such as mammary gland was associated with spontaneous development of mammary intraepithelial neoplasia and invasive mammary carcinomas (Artandi et al., 2002). This suggested that *TERT* expression could promote the development of spontaneous cancer in mice with wild-type (WT) telomere length (Artandi et al., 2002). In another experiment using the GM847 cell line that maintains its telomere by ALT, expression of oncogenic RAS failed to fully transform the cells. Nonetheless, these cells acquired a tumorigenic phenotype upon ectopic expression of *TERT* suggesting that TERT had an additional function required for cellular transformation independent of its ability to maintain telomeres (Stewart et al., 2002). Along the same line, ectopic expression of telomerase in primary human mammary epithelial cells (HMECs) identified an apparently telomere-independent function of hTERT that allowed HMECs to proliferate in mitogen-deficient conditions through the induction of several growth-promoting genes, including the epidermal growth factor receptor (EGFR) (Smith et al., 2003). Moreover, transcriptional profiling of normal and TERT-immortalized fibroblasts identified 172 differentially expressed genes in TERT-immortalized cells, one of them epiregulin being a potent growth factor associated with cancer. These results suggested that both activation of telomerase and subsequent induction of epiregulin were required to sustain cell proliferation (Lindvall et al., 2003). These observations, therefore, suggested that telomerase activation promotes tumorigenesis and immortalization independently of its essential function in telomere elongation.

Despite pieces of evidence indicating telomerase had extratelomeric roles, some controversies persisted in the field. In a landmark paper (Sarin et al., 2005), *mTERT* was expressed under the control of a tetracycline responsive promoter allowing the conditional expression of *mTERT*. Activation of mTERT in mice (that were otherwise WT for the endogenous telomerase) promoted the proliferation of hair follicle bulge cells. Importantly, the effects related to this artificially induced expression of *mTERT* did not require the telomerase RNA component (Sarin et al., 2005) and was proficient in a telomerase mutant carrying a point mutation abolishing telomerase catalytic activity (Choi et al., 2008). These data provided an *in vivo* evidence that ectopically expressed mouse telomerase, likely above a certain level, promoted proliferation of resting cells. As an ectopic expression of telomerase did not reflect a physiological situation, it remains, however, to be determined whether it could be relevant in the context of cancer cells (that are known to reactivate telomerase expression). Therefore, the question arises about whether these extratelomeric functions of TERT operate in cancer cells, and above which threshold. It was further shown by the same group that mTERT interacted with BRG1, the central catalytic subunit of numerous chromatin-modifying enzymatic complexes (Trotter and Archer, 2008) either in co-immunoprecipitation with ES cells or *in vitro* in pull-down experiments (Park et al., 2009). Because BRG1 interacts with members of the Wnt signaling pathway (Barker et al., 2001) whose stimulation activates quiescent epidermal stem cells, and also because transcriptomic profiling unveiled similarities between TERT and Wnt transcriptional responses (Choi et al., 2008), Park et al. (2009) tested whether mTERT could activate the Wnt pathway *ex vivo* (in cells depleted or not for BRG1) and *in vivo* by inducing the expression of *mTERT* placed under the control of a tetracycline regulated promoter. They measured Wnt signaling in the gastrointestinal tract using mice engineered with a Wnt-reporter (Park et al., 2009). Collectively, their data showed that *mTERT* overexpression stimulated the transcription of targets of the Wnt/β-catenin pathway *in vitro* in a BRG1-dependent way and *in vivo* in progenitors of the small intestine (Park et al., 2009). Again, the question related to this work was whether mTERT could activate the Wnt pathway in a WT mouse. Strikingly, they observed that 50% of G1 *mTERT*^–/–^ mice exhibited homeotic transformation of the vertebrate, resembling the one observed in mice carrying a hypomorphic mutation of WNT3A, while none of the WT counterparts showed this abnormality (Park et al., 2009). The authors elegantly proposed that stimulation of progenitor cells by the Wnt pathway should somehow be coordinated to telomere maintenance and such coupling could have led to the incorporation of telomerase as a co-factor of β-catenin in the Wnt signaling pathway (Park et al., 2009). Finally, the same group showed in mouse that conditional expression of *mTERT* or a catalytically dead mTERT in the kidney induced podocyte proliferation and resulted in collapsing glomerulopathy (Shkreli et al., 2011). Consistent with their previous results, Wnt signaling was activated in the podocytes that re-enter the cell-cycle suggesting that Wnt signaling could underline podocyte activation upon *mTERT* expression.

However, these conclusions were questioned in several independent articles. By analyzing phenotypes of heterozygous *mTERT^+/–^* mice in backgrounds with different telomere length settings, Strong et al. (2011) reported that *mTERT^+/–^* and *mTERT*^–/–^ displayed similar phenotypes with progressive breeding that correlated with telomere shortening. Moreover, they found that in the CAST/EiJ genetic background (short telomere setting), *mTERT* and *mTR* deficiencies produced indistinguishable phenotypes. In addition, Wnt was activated similarly in WT or *mTERT*^–/–^ cells (Strong et al., 2011). These results strengthened the admitted notion that short telomeres, and not the absence of telomerase, are at the root of tissue renewal limitation. Of note, these results were not really in contradiction with the results from Park et al. (2009) since the latter mainly investigate the effects of ectopic/overexpression of telomerase and not its absence. The only apparent discrepancy between the two studies was the fact that Strong et al. (2011) could not find abnormal ribs in *mTERT*^–/–^ mice in contrast to what was observed by Park et al. (2009), when they examined homeotic transformations in G1 *TERT*^–/–^ mice. To explain this discrepancy, it was suggested that mTERT function in regulating Wnt could be compensated in the germline of telomerase knockout mice.

In a second article, Listerman et al. (2014) analyzed the ability of hTERT to modulate Wnt signaling in several breast cancer cell lines endowed with different levels of telomerase activity. In breast cancer cell lines expressing a high level of telomerase, additional overexpression of *hTERT* did not activate Wnt signaling reporters while a mild activation was observed in a cell line that was originally endowed with mild-telomerase activity. The authors concluded that hTERT did not stimulate Wnt target transcription universally but instead did so in a context-dependent way. These results did not support those from Park et al. (2009) but they were not strictly contradictory. More annoying was the questioning of the interaction between hTERT and BRG1. However, both co-immunoprecipitations were done in different contexts. While Park et al. (2009) immunoprecipitated endogenous HA-tagged mTERT (tagged at its locus) in mouse ES-cells and Listerman et al. (2014) analyzed the interaction between overproduced FLAG-hTERT and BRG1 in HeLa cells. Again, the context was different. Of note, in an independent study, ectopically expressed FLAG-hTERT isolated from HeLa cells was shown to interact with endogenous BRG1 and nucleostemin. This complex was shown to assemble during mitosis and to play a role in the maintenance of heterochromatin at repetitive elements found at centromeres and retrotransposons (Maida et al., 2014). Noteworthy, nucleostemin is involved in telomere protection through the regulation of TRF1 sumoylation and its subsequent association with the promyelocytic leukemia protein isoform IV (PMLIV) in telomerase-negative cells (Hsu et al., 2012). Finally, BRG1 and p54 (nrb) were reported to cooperate in regulating *TERT* splicing and promoting the production of full-length *TERT* mRNA. This action seems independent of its interaction with β-catenin (Ito et al., 2008).

It turned out that in a more recent article, late-generation *hTR^–/–^* mice were shown to exhibit downregulation of either factor involved of the canonical WNT signaling, or targets of WNT pathway in the intestinal crypt epithelia (Yang et al., 2017). Pharmacological treatments that restored expression of components of the WNT pathway as well as one of the WNT targets in the epithelium of *hTR^–/–^* also restored telomere capping in the mutant epithelium. Importantly, this improved telomere capping was explained by the restoration of shelterin protein expression, including TRF2 and Pot1a rather than by telomere lengthening (Yang et al., 2017). This result was consistent with a previous study showing that TRF2 was transcriptionally activated by the Wnt/β-catenin signaling pathway in mouse intestinal tissues (Diala et al., 2013). Given that telomerase expression is also positively regulated by Wnt in the context of stem cells and cancer (Hoffmeyer et al., 2012), the results indicate that the Wnt signaling pathway, telomerase expression, and telomere capping by shelterin proteins mutually reinforce themselves through a positive feedback loop (Yang et al., 2017). In the light of these results, the same team proposed that the developmental transformations in the axial skeleton that were observed in G1 *mTERT^–/–^* could be due to the presence of one or a few numbers of single aberrantly shortened telomere in the germline of the parents *TERT^+/–^* that were used to create the G1 *TERT*^–/–^ mice (Fernandez and Johnson, 2018). Whether one or a few uncapped telomeres could downregulate the canonical WNT signaling pathway remains an open question.

### TERT and NF-κB

The first evidence indicating that TERT regulates NF-κB-dependent gene expression was reported in multiple myeloma cells in which NF-κBp65 subunit was found to modulate the nuclear translocation of TERT (Akiyama et al., 2003). Ten years later, it was reported in another landmark paper that TERT stimulated the expression of a number of genes whose transcription is controlled by the NF-κB pathway such as IL6, IL8, and tumor necrosis factor-α (TNFα) (Ghosh et al., 2012). This result was reinforced by the observation that human TERT regulates MMP expression *via* NF-κB-dependent transcription independently of TERT telomere elongation activity (Ding et al., 2013). Several evidences indicated that TERT regulates NF-κB-dependent gene expression by binding to the NF-κB p65 subunit although no evidence of a direct protein/protein interaction was provided (Ghosh et al., 2012). Because *TERT* transcription can be stimulated *ex vivo* by expressing NF-κB (Yin et al., 2000), it was proposed a “feed-forward” regulation between TERT and NF-κB (Li and Tergaonkar, 2014). Since NF-κB is a key regulator of inflammatory and developmental processes, hTERT is likely to regulate inflammation and development through its interplay with NF-κB.

### TERT and MYC

Whereas MYC has been described mostly as a direct activator of TERT transcription by binding to the E-box (5′-CACGYG-3′) in the *TERT* promoter region (Greenberg et al., 1999; Wu et al., 1999), it appears that TERT also regulates c-Myc. Indeed, recent results indicate that first-generation TERT-null mice exhibit a delayed onset of MYC-induced lymphomagenesis in a lymphoma murine model (Koh et al., 2015). The authors further showed that TERT stabilizes MYC protein levels and regulates its binding to target promoters contributing to either activation or repression of MYC-target genes, independently of its telomeric role. These results unveil another example of feed-forward mechanism potentializing MYC-dependent oncogenesis.

### TERT, Sp1, and VEGF

Vascular endothelial growth factor (VEGF) is another example of a factor whose expression is regulated by TERT. TERT was first reported to activate the transcription of VEGF in embryonic lung cells and HeLa cells (Zhou et al., 2009). In support of this finding Liu et al. (2016) observed that in tumor xenograft assays of lung carcinoma carried out in WT or G1 *TERT* knockout mice (without detectable telomere dysfunction), tumor development as well as VEGF expression were reduced in *TERT*^–/–^ mice compared to WT suggesting that TERT deficiency affects tumorigenesis and tumor vascular development (Liu et al., 2016). Mechanistically, this activation may occur through hTERT binding to the transcription factor Sp1 at the *VEGF* promoter in order to stimulate angiogenesis (Liu et al., 2016).

### TERT and DNMT

In 2003 was published the first study reporting a novel function of hTERT in the regulation of DNA methyltransferases (DNMTs) (Young et al., 2003). Telomerase introduction in normal human fibroblasts upregulates the expression and the activity of the DNMT1. DNMT1 activity is to maintain the pattern of DNA methylation. Therefore, even though the precise mechanism was not elucidated, the consequence of this increase is the prevention of changes in gene expression related to cellular aging. Recently, by exploring the correlation between hTERT and DNMT3B (DNMTs 3B) in both hepatocarcinoma (HCC) cell lines and primary HCC tumors, Yu et al. (2018) reported that TERT up-regulated DNMT3B expression. This DNMTs that is required for *de novo* methylation of CpG dinucleotides cooperated with Sp1 to activate DNMT3B promoter activity. Indeed, Sp1 inhibition significantly attenuated TERT-mediated DNMT3B up-regulation, while its over-expression restored DNMT3B expression in TERT-depleted cancer cells (Yu et al., 2018). One of the consequences of TERT-induced DNMT3B up-regulation was the aberrant methylation of the tumor suppressor PTEN promoter and its subsequent silencing. As PTEN inhibits PI3K/AKT signaling, TERT-mediated PTEN silencing led to an increase of AKT activity which in turn promoted cell survival and proliferation providing another example of the role of TERT in carcinogenesis.

### TERT, Chromatin Remodeling, and the DNA Damage Response

Physical protection of telomeres by capping may explain in some cases the prevention of telomere damages due to oxidative stress (as reported in Fanconi Anemia) (Uziel et al., 2008). An additional function of TERT in modulating the DNA damage response (DDR) was proposed by Masutomi et al. (2005) who reported that the down-regulation of hTERT reduced the level of H2AX phosphorylation and ATM autophosphorylation in fibroblasts exposed to DNA damaging agents while telomere length was unchanged. They proposed that the sustained loss of hTERT expression compromised the DDR through an alteration of chromatin structure. Recently, it was discovered that small non-coding RNAs (dilncRNA) were produced at DNA double-strand breaks that were critical for DDR activation (Michelini et al., 2017). It is tempting to speculate that the effect mediated by TERT on the DDR might be due to interference between TERT and the generation of the dilncRNA.

## Tert, ROS, and Mitochondria: An “Odd MÉNage À Trois”

Many other articles reported in different cellular systems that telomerase was protecting against oxidative stress and that this protection was associated with reduced levels of reactive oxygen species (ROS) (Armstrong et al., 2005; Ahmed et al., 2008; Haendeler and Klotz, 2008; Indran et al., 2011; Mattiussi et al., 2012; Spilsbury et al., 2015). However, opposing findings complicated the understanding of the interplay between telomerase ectopic expression and oxidative stress. Several reports initially showed that hTERT was found not only in the nucleus but also in the cytoplasm and mitochondria (Haendeler et al., 2003; Santos et al., 2004; Sharma et al., 2012). The move out of hTERT from the nucleus into the mitochondria was in particular triggered by oxidative stress (Santos et al., 2006; Ahmed et al., 2008; Singhapol et al., 2013). It was shown in human fibroblasts that the accumulation of overexpressed TERT in mitochondria under oxidative stress protected mitochondria DNA (mtDNA) against acute or oxidative damage and decreased superoxide production and cellular ROS levels (Ahmed et al., 2008). In an independent study, it was shown that an overexpressed C-terminus Myc-tagged TERT was localized into the mitochondrial matrix consistent with the fact that TERT has a matrix-targeting sequence at its N-terminus (Haendeler et al., 2009). In the same study, it was shown by chromatin immunoprecipitation that TERT-Myc bound two mtDNA regions called ND1 and ND2 (NADH ubiquinone oxidoreductase subunits 1 and 2) of complex I and protected mtDNA from UV-light damage. However, this result was challenged by the finding that hTERT rather binds to mtDNA non-specifically or through a widely distributed *cis*-element (Sharma et al., 2012). Haendeler et al. (2009) further showed that inactivating the catalytic activity of TERT reduced by 30% the respiratory chain activity of TERT-transfected cells suggesting that TERT catalytic activity was required to protect the respiratory chain. They proposed that TERT under certain circumstances increased the respiratory chain activity through complex I (Haendeler et al., 2009; Ale-Agha et al., 2014). Related studies indicated that mitochondrial hTERT worked as a RT with tRNAs or other RNAs involved in mtDNA replication but not with *hTR* (Sharma et al., 2012). Whether hTERT contributes to replication or repair of mtDNA during oxidative stress and how its RT activity contributes to this process remains unknown. Further work is required to directly link the mitochondrial action of hTERT to its mitochondria protective effect.

Oddly, the mitochondria protective effect of hTERT was opposite to the one reported by Santos et al. (2004, 2006) few years ago in which ectopic expression of hTERT in response to H_2_O_2_ resulted in higher levels of mtDNA damage and increase in apoptotic cell death in a way that required the hTERT catalytic activity (Santos et al., 2004, 2006). It may be that expressing TERT at different levels differentially regulate signaling pathway as described above raising the possibility that different effects of TERT overlap to produce the observed phenotypes. The same authors recently reported a differential activation of autophagy in human fibroblasts according to the subcellular localization of TERT in response to H_2_O_2_. They proposed that the expression of an hTERT mutant lacking mitochondrial localization exhibited increased autophagy as a rescue pathway to compensate for the loss of hTERT in mitochondria (Green et al., 2019).

Beyond the mtDNA centric view of hTERT action, other explanations were provided to account for reduced endogen ROS production in response to oxidative stress. The effect of hTERT would result from the combined effects of nuclear and mitochondrial hTERT. Within the nucleus, the modulation of signaling pathways by hTERT (such as the NF-κB pathway) would contribute to antioxidant hTERT effect by promoting the transcription of antioxidant factors including MnSOD and g-glutamylcysteine (Indran et al., 2011). In mitochondria, the imported hTERT would improve the mitochondrial activity as illustrated by the higher activity of cytochrome C oxidase (Indran et al., 2011). Related to this study, the impact of hTERT was evaluated in response to TNF-α, an activator of NF-κB signaling pathway and a powerful inducer of endogenous ROS (Mattiussi et al., 2012). It was shown that hTERT overexpression in human foreskin fibroblasts repressed ROS-dependent activation of ERK1/2 protein kinases and of superoxide dismutase 2 (SOD2) providing additional insights that hTERT represses ROS-dependent intracellular signaling (Mattiussi et al., 2012). A recent study also identifies a BH3-like motif in hTERT protein (Jin et al., 2018). This motif is found in most Bcl2 family proteins. The same study reports an interaction between hTERT and two anti-apoptotic Bcl2 protein family, Mcl1 and Bcl-xL, and also with BECN1, a BH3-only protein involved in autophagy supporting another functional link between hTERT and the mitochondria biology.

All these results indicate intricate links between telomerase, ROS, and mitochondria. However, many of these results were obtained in cellular systems designed to strongly overproduce hTERT. The question remains about the relevance of the anti-oxidative function of hTERT during development or in response to stresses in adult in specific tissues or in cancer cells. Recent results indicate that the protein TERT persists in the adult brain while telomerase activity was shut-down before birth (Ulaner et al., 1998; Iannilli et al., 2013; Ishaq et al., 2016; Miwa et al., 2016; Miwa and Saretzki, 2017). In mouse, TERT is expressed in several regions of the adult brain such as the olfactory bulb, hippocampus, cortex, and cerebellum (Caporaso et al., 2003; Flanary and Streit, 2003; Eitan et al., 2012). Interestingly, in mouse Purkinje neurons, TERT localization to mitochondria was increased after exposure to X-ray radiation or to high glutamate levels suggesting that telomerase could have specific roles in protecting neurons exposed to excitotoxic and genotoxic stresses (Eitan et al., 2016). Further work using animal models will be required to confirm the relevance of these fascinating observations.

## What Can Explain the Discrepancies in the Results Obtained Regarding the Extratelomeric Functions of Telomerase

Although much is going to be learned upon these new telomere unrelated roles of telomerase, studies may generate different interpretations because of the multiplicity of the models and the lack of accurate tools to detect the endogenous TERT and measure its activity. This can explain some of the discrepancies in the published results pointing out that alternative approaches have to be taken to quantify the level of TERT, determine its cellular localization, or identify its partners. Overall, there is a need to develop appropriate experimental tools to go further in the elucidation of the TERT functions in normal physiology and the context of tumors.

### In Cancer Cells, hTERT Is a Lowly Expressed Protein With Only Several Hundred Molecules per Cell

Despite the presence of telomerase activity in cancer cells, the mRNA abundance is low compared to other genes even in tumor cells (Yi et al., 2001; Xi and Cech, 2014) making it difficult to detect and quantify accurately and this represents a major obstacle. Telomerase activity is currently measured using a PCR-based assay (telomerase repeated amplification protocol or TRAP assay), which is very sensitive but not very accurate and susceptible to artifacts. Experiments based on *TERT* overexpression (either WT or mutant variants) have to be taken with caution since the levels obtained are generally much more important than those observed in tumor cells. Indeed, overexpression can alter hTERT localization (high expression in both nuclei and cytoplasm) and the recruitment to telomeres or give rise to artificial protein interactions. Furthermore, *TERT* overexpression in telomerase positive cells can affect telomere length. It is now documented that telomere length can control some gene expression near the ends of the chromosome either by telomere position effect (TPE) or by TPE over long distances (TPE-OLD) (Baur et al., 2001; Koering et al., 2002; Kim et al., 2016). Therefore, *TERT* overexpression may modulate gene expression by these processes. This modulation, therefore, occurs indirectly through a telomere-dependent mechanism. Of note, the human *TERT* gene is located at the very end of chromosome 5, near the telomere. Therefore, TPE-OLD mechanisms could be effective mechanisms in TERT gene regulation. In contrast, in mice *TERT* gene is on chromosome 13 and the telomeric location is not conserved. Consequently, the local genomic structure around *TERT* locus is quite different between mice and humans. These observations suggest that different strategies for *TERT* regulation have evolved between human and mice. Therefore, although mouse models prove to be extremely useful to unravel telomerase functions, we have to be aware that many of them do not systematically mimic human diseases, including cancers, and caution must be used in drawing definitive conclusions.

### Commercially Available TERT Antibodies Are Either Inefficient or Non-specific in Targeting Endogenous TERT

Reliable detection of the endogenous TERT protein has proven difficult due to the low abundance of TERT protein and the lack of available specific antibodies (Wu et al., 2006). Several experiments demonstrating interaction involving TERT protein rely mainly on immunoprecipitation using TERT antibodies that were not properly validated. Another concern is that in several experiments tags have been fused to TERT to detect the presence of the protein or its activity. However, it has to be checked whether the modifications introduced in the TERT protein does not modify its functions. Indeed, it has been shown, for example, that C-terminal tagging impairs the ability of telomerase to elongate telomeres within cells even though the activity of the protein measured *in vitro* was retained (Banik et al., 2002).

### Alternative Splicing Variants of hTERT

Among the mechanisms involved in TERT regulation, one is alternative splicing. To date, 22 and 31 splice variants of *hTERT* have been described in human and in chicken, respectively (Kilian et al., 1997; Wick et al., 1999; Hisatomi et al., 2003; Chang and Delany, 2006; Saeboe-Larssen et al., 2006; Amor et al., 2010). As most of these variants are disrupted in the major functional domains of the TERT protein, they may result in an inactive telomerase complex in terms of telomere elongation. However, accumulating evidence points to potential functions for some of these variants. For example, a human variant, containing an in-frame deletion that removed exons 4–13 (Δ4–13) and encoding the catalytic domain of telomerase was described (Hrdlickova et al., 2012). This variant (that produces a truncated protein) was expressed in telomerase-negative as well as in telomerase-positive cell lines (Hrdlickova et al., 2012). Importantly, its overexpression leads to elevated proliferation rates of several cell types by stimulation of Wnt signaling. Note that another study of splice variants reported that the deletion variant lacking most of the RT domain is highly expressed in stem and cancer cells. It inhibits telomerase activity and confers a growth advantage to breast cancer cells independent of telomere maintenance, by inhibiting cisplatin-induced apoptosis (Listerman et al., 2013). It is known that alternative splicing is deregulated in cancers leading to the expression of splice variants detected during development but silenced in normal cells. Therefore, many cancers show changes in *TERT* splicing patterns. As *TERT* splicing plays a major role in dictating the activity of telomerase at the telomeres, it remains to be assessed whether these splice variants have other non-conventional activities. The fact that many non-canonical functions of full-length *TERT* do not require its catalytic activity support evidence for possible functions of alternatively spliced *TERT* isoforms. This notion is further corroborated by the study using a panel of *TERT* mutants transduced into HMECs, which shows that TERT had biological actions in cell proliferation and DNA damage signaling that could be genetically separated and functionally independent (Mukherjee et al., 2011).

The existence of the *TERT* splice variants may complicate greatly the interpretation of expression data as these are generally not taken into account in the quantification of *TERT* mRNA levels because of technical and methodological limitations to quantify their expression. The most common assay to measure *hTERT* expression is quantitative RT-PCR using primers designed to detect exons 5–9 localized in the catalytic RT domain. However, these exons are precisely belonging to those subjected to splicing, leading to misinterpretation of the data regarding the expression of *hTERT*. Furthermore, depending on the cell line or tissue studied, the amount of the full-length protein coding *TERT* RNA can represent 1–90% of all the *TERT* transcripts (Yi et al., 2001). It is also important to note that alternative splicing of *TERT* is not the same in human and mice (Sayed et al., 2019). The major transcript in mice for *TERT* is the full-length transcript whereas in humans most *TERT* transcripts are alternatively spliced to variants incompetent for the elongation of telomeres. However, these variants may have conserved the alternative functions of TERT or even novel functions that remained to be identified. Of note, certain normal cells have been observed to express “non-functional” transcripts (Fan et al., 2005; Li et al., 2008; Wong et al., 2014). The physiological significance of this observation remains unclear but support that these variants could have a role in cell physiology. As previously mentioned, the poor availability of TERT antibody and the lack of reliable methods for the quantification of each isoform variant transcript hamper the functional characterization of these variants. Indeed, the most common assays to measure *hTERT* transcripts are designed to detect exons 5–9 in the RT domain. However, since in human these exons are spliced out in most transcripts, the conclusions drawn may have been misinterpreted. In a recent study, it was reported that *TERT* transcript are expressed in normal cell and tissues at a level similar to the one measured in cancer cells suggesting that the major difference in telomerase activity between normal and cancer cells relies in a shift in splicing (Kim et al., 2016). Emerging sequencing technologies will prove helpful to quantify splice variants. Finally, it is also important to note that *TERT* alternative splicing in the mouse is not the same as in human (Sayed et al., 2019). Indeed, in mouse cells the majority of *TERT* transcripts contain the RT domain able to produce an active TERT protein while in humans the RT domain of human *TERT* is spliced out in most of the transcripts.

## What Are the Consequences of the Existence of hTERT Telomere-Unrelated Functions for the Development of Anticancer Strategies?

As pointed out, an accumulation of evidence indicates that TERT plays important functions far from telomere maintenance. These functions can impact cancer cells by giving them a selective advantage. Presently, most telomerase inhibitors designed to inhibit telomerase activity (i.e., synthesis of the telomeric repeats) are not well tolerated by the patients. Indeed, therapy must be prolonged because it could take a long period before telomeres are shortened enough to induce death of the cancer cell. To be fully efficient, it could be necessary to target different functions of hTERT instead of solely those that inhibit its enzymatic activity or accessibility to telomeres. In this case, therapeutic strategies targeting directly hTERT expression will be highly effective and more prompt as it will target all TERT functions. In this line of view, it has been shown that retinoic acid can inhibit the transcription of hTERT leading to cell death. The induction of the death can be the result of telomere shortening but it has been also demonstrated that the repression of hTERT can sensitize cells to conventional chemotherapy in a telomere independent manner (Dudognon et al., 2004; Deville et al., 2011; Samy et al., 2012). Indeed TERT can increase resistance to chemotherapeutic agents. These results encourage the integration of strategies targeting the expression of *hTERT* (as retinoic acid) into extended trials performed *in vitro* and clinical practice. Besides, it is clear that in some cases as telomerase is associated with many extratelomeric functions, total inhibition of its expression could induce severe and unpredictable side effects. It is now clear that the therapeutic exploitation of the biology of the telomeres requires the integration of the multiple roles of telomerase in the physiology of not only cancer cells but also normal cells. Therefore, to develop new strategies of telomerase inhibition for anticancer therapy, a more thorough understanding of all the functions of the telomerase complex (conventional and alternative) and the interplay between these functions in the physiology of the cell is necessary.

## Conclusion

Telomerase has been described as having a key role in several signaling pathways that are frequently altered in cancers. The major role of hTERT in cancer has been related to the maintenance of telomere length that is a prerequisite event for the tumor cells to survive and proliferate. However, telomere unrelated functions have emerged to allow increased proliferation, resistance to cell death, inflammation, invasion, and metastasis. Therefore, endowed with these non-telomeric functions, TERT can participate to all the major characteristics of the cancer phenotype described by Hanahan and Weinberg (2000, 2011). However, as pointed out in this review, the complexity of the interconnecting signaling networks and feed-forward responses illustrates the difficulty of drawing mechanistic conclusions.

## Author Contributions

Both authors contributed equally to the work and approved it for publication.

## Conflict of Interest

The authors declare that the research was conducted in the absence of any commercial or financial relationships that could be construed as a potential conflict of interest.
